# Variability in Definitions and Criteria of Extrauterine Growth Restriction and Its Association with Neurodevelopmental Outcomes in Preterm Infants: A Narrative Review

**DOI:** 10.3390/nu16070968

**Published:** 2024-03-27

**Authors:** Clara González-López, Gonzalo Solís-Sánchez, Sonia Lareu-Vidal, Laura Mantecón-Fernández, Aleida Ibáñez-Fernández, Ana Rubio-Granda, Marta Suárez-Rodríguez

**Affiliations:** 1 Unidad de Neonatología, Área de Gestión Clínica de Pediatría, Hospital Universitario Central de Asturias, 33011 Oviedo, Spain; claragonlo93@gmail.com (C.G.-L.); sonia.lareu.vidal@gmail.com (S.L.-V.); laura_mantecon@hotmail.com (L.M.-F.); maleidaib@hotmail.com (A.I.-F.); anarg59@gmail.com (A.R.-G.); msr1070@hotmail.com (M.S.-R.); 2 Instituto Investigación Sanitaria Principado de Asturias, ISPA, 33011 Oviedo, Spain; 3 Medical Department, University of Oviedo, 33003 Oviedo, Spain

**Keywords:** extrauterine growth restriction, neurodevelopment, prematurity

## Abstract

Extrauterine growth restriction (EUGR) has been used in the literature and clinical practice to describe inadequate growth in preterm infants. Significant variability is seen in the criteria for EUGR, with no standard definition reached to date. Moreover, no consensus on the optimal timing for assessment or the ideal growth monitoring tool has been achieved, and an ongoing debate persists on the appropriate terminology to express poor postnatal growth. To ensure an adequate understanding of growth and early intervention in preterm infants at higher risk, it is critical to relate the diagnostic criteria of EUGR to the ability to predict adverse outcomes, such as neurodevelopmental outcomes. This narrative review was conducted to present evidence that evaluates neurodevelopmental outcomes in preterm infants with EUGR, comparing separately the different definitions of this concept by weight (cross-sectional, longitudinal and “true” EUGR). In this article, we highlight the challenges of comparing various published studies on the subject, even when subclassifying by the definition of EUGR, due to the significant variability on the criteria used for each definition and for the evaluation of neurodevelopmental outcomes in different papers. This heterogeneity compromises the obtention of a single firm conclusion on the relation between different definitions of EUGR and adverse neurodevelopmental outcomes.

## 1. Introduction

### 1.1. Growth in the Preterm Infant

The postnatal growth of preterm infants continues to be a challenge in neonatology [1]. A consensus among neonatologists on the ideal growth pattern for preterm infants and on the optimal practices for the monitoring of growth in the neonatal intensive care unit (NICU) has yet to be reached [2].

Traditionally, growth in the preterm infant has been described to aim to mimic the pattern of intrauterine life, as published by the American Academy of Pediatrics (AAP) in 1977 [3]. The World Health Organization (WHO) estimates that weight gain during fetal life is 20–23 g/kg/day between 23 and 25 weeks of gestation, 17–20 g/kg/day from 26 to 29 weeks and 10–13 g/kg/day between 35 and 37 weeks [4]. 

However, this pattern of fetal growth for the preterm has been questioned, as some differences have been noted between growth in the preterm infant and expected fetal growth. Firstly, it has been reported that most preterm infants born between 24 and 29 weeks of gestation might not achieve the median birth weight of the same reference fetus at hospital discharge [5]. Secondly, the physiological weight loss that occurs in the first days of life, followed by a subsequent birth weight recovery, seems to differ in preterm infants depending on the gestational age compared to term newborns. In a study published in 2016 that evaluated the postnatal growth in preterms considered “healthy” (based on minimal support for the gestational age), the maximum weight loss was noted to be 11% on day 5 of life for infants born between 25 and 29 weeks of gestational age and 7% for infants delivered between 30 and 34 weeks of gestational age [6]. The most recent guidelines from the European Society for Paediatric Gastroenterology Hepatology and Nutrition (ESPGHAN) recommend nutritional strategies for infants admitted in the NICU aiming to achieve birth weight recovery between day 7 and 10 of life in the preterm population [7]. Thirdly, it is unclear whether preterm infants truly follow fetal growth charts. Studies have described that preterm infants follow a weight curve lower than the percentile at birth after the initial weight loss [6]. 

### 1.2. Growth Assessment in the NICU and Extrauterine Growth Restriction

An important challenge for the study of the growth in the preterm infant arises from the variability of strategies and tools available for growth assessment in the NICU. Multiple methods have been described for evaluating growth in preterm infants [8]. The most frequently used include absolute anthropometry measurements and their conversion to reference charts or standards of reference [9]. Moreover, different reference charts are available for the calculation of percentiles and z-scores in preterm infants, such as Olsen [10], Bertino [11] and Fenton [12]. These reference charts describe the growth of the specific population studied. On the contrary, WHO [13] and INTERGROWTH-21 [14] were proposed as standards of growth, with the goal to describe how infants should grow under optimal conditions. Most of the available growth charts are national-based, considering they were developed for the study of a specific population. Consideration should be made to the international scope of two growth charts. INTERGROWTH-21st included infants from eight countries (Brazil, Italy, China, India, the United Kingdom, Kenya, Oman and the United States), and Fenton is based on a systematic review that included six studies with infants from Germany, Australia, Canada, the United States, Scotland and Italy.

Growth velocity is also frequently used in daily practice and reported in the literature as a tool for monitoring an infant’s growth. Nevertheless, significant variability has also been reported in the formulas used for estimating growth velocity [15].

There are some particularities of the preterm population that make difficult the establishment of an optimal tool for growth evaluation in those infants. Firstly, some of the growth references previously named are based on cross-sectional data of infants at birth. However, growth in the preterm infants admitted to the NICU has been described to differ from expected fetal growth as previously presented. Furthermore, it is difficult to create standards of postnatal growth in this population due to the different comorbidities that can be experienced by preterm infants that compromise the establishment of a “healthy” preterm [16]. Additionally, extrauterine growth restriction (EUGR) and postnatal growth failure (PGF) have been described in the literature to identify inadequate postnatal growth not meeting the expectations [17]. There is significant variability in the criteria, definitions and timing for diagnosis for EUGR both in the literature and in clinical practice.

Traditionally, EUGR has been described as a weight below the 10th percentile [18], <−2 Z-score [19], <−1.5 z-score [20] or, less frequently, below the 3rd percentile [21] using different growth charts (referred to as the cross-sectional definition). An alternate longitudinal definition has been applied to weight loss more than one [21] or two standard [19] deviations from the weight at birth. Recently, new criteria have been proposed. These new approaches, named “true” cross-sectional and “true” longitudinal EUGR, include patients not small for their gestational age at birth who meet criteria for EUGR with the previously described definitions, respectively [22,23]. Furthermore, EUGR has been defined with variable timing for diagnosis. The most frequent criteria range from 36 weeks of corrected gestational age to discharge from the hospital.

A systematic review conducted in 2017 by Fenton et al. [24] reported that nineteen percent of published preterm infant growth studies described rates of EUGR with significant differences both in the definition and the timing of assessment. A total of 62 percent of these studies used a tenth percentile cut-off, 10% used third percentile cut-off and 18% used losses of a 2 z-score compared to birth. Moreover, 63% of the studies conducted EUGR assessment at discharge, 21% at term age and 20% at 36 weeks.

Despite the described frequency of the use of the term EUGR, controversies surrounding this concept have also been raised. A group of experts published in 2020 an article highlighting the potential harms associated with over-diagnosing growth deviation with EUGR and PGF [25]. Among the concerns pointed out in the article, they described that EUGR is usually defined solely by weight with no consideration to length nor head circumference, fails to recognize postnatal weight loss and the subsequent growth pattern, is not clearly related to neurodevelopmental outcomes and is usually based on an arbitrary statistical cut-off. Moreover, Fenton et al. [25] also proposed that various cut-off criteria for EUGR should be examined, calculating the diagnostic accuracy for important outcomes such as neurodevelopment. This constitutes a field of promising and interesting future studies.

## 2. Materials and Methods

The primary objective of this study is to review the literature that evaluates neurodevelopmental outcomes in preterm infants with extrauterine growth restriction defined by weight. Given the variability in criteria for this concept previously described, studies were grouped by the different definitions of EUGR analyzed (cross-sectional, longitudinal, “true” cross-sectional and “true” longitudinal EUGR).

Studies were selected according to the outlined criteria. The search strategy included all types of studies with the exclusion of case series and case reports.

We included studies examining preterm infants (gestational age less than 37 weeks) with extrauterine growth restriction by weight, including all definitions of this concept previously described. Studies addressing both preterm and term infants, if the data provided for preterm infants were reported separately, were also included for the search. Neurodevelopmental outcomes (cerebral palsy, any psychomotor developmental indices, school readiness and school performance) were the outcomes of interest. There were no restrictions on the length of follow up or on the type of setting. A language restriction was not placed on the literature search, although to sufficiently assess the quality of each study, we required the full text to be available in English, French or Spanish. Literature search strategies were developed using Medical Subject Headings (MeSH) and text words related to prematurity and EUGR. MEDLINE was searched using the described criteria.

## 3. EUGR and Neurodevelopmental Outcomes

**Figure 1:**
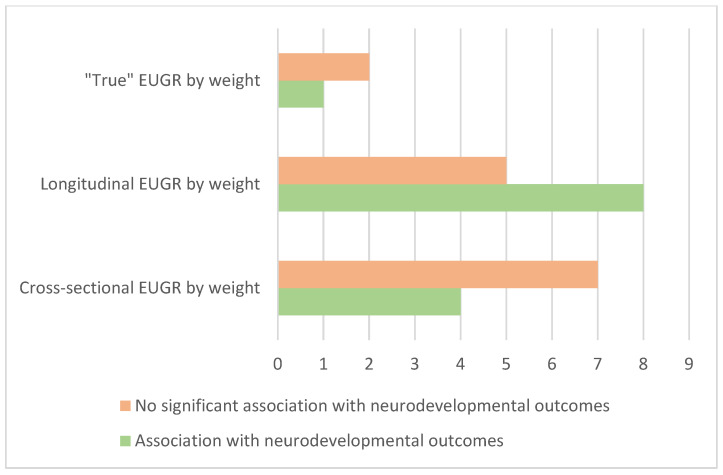
A bar graph of the number of studies included in the review describing the association between EUGR by weight and neurodevelopmental outcomes, divided by the EUGR definition.

Numerous studies have examined the risk of neurodevelopmental impairment in preterm infants and its relation to EUGR. A systematic review, published in 2020 studying comorbidities experienced by children with a neonatal diagnosis of EUGR, reported that EUGR was associated with poorer neurodevelopment [26]. However, it is important to note that the variability in the definitions for EUGR and the heterogeneity in the evaluation of neurodevelopmental outcomes complicate comparisons between the results obtained in the different studies. Accordingly, controversy persists regarding the association of extrauterine growth restriction and its criteria changes and neurodevelopment. In an effort to understand the literature, we will detail it based on the EUGR definition. Figure 1 presents a graphical summary of the studies included reporting positive and no significant association between EUGR by weight and worse neurodevelopmental outcomes.

### 3.1. Cross-Sectional EUGR and Neurodevelopmental Outcomes

Several studies have assessed the impact of EUGR on neurodevelopment using the cross-sectional definition in its different modalities (weight less than a variable percentile or z-score cut-off at a specific point in time) with discrepancies in the results as summarized in Table 1.

From the reviewed articles, for EUGR defined by a weight less than the 10th percentile, some did not show a significant association with poor neurodevelopmental outcomes at 24 months of postmenstrual age [21,36] whilst others did [38]. Shah et al. [21] assessed neurodevelopment in preterm infants with a gestational age of less than 28 weeks using Bayley Scales of Infant Development, Second Edition (BSID-II) at 18–24 months. They found no significant association with neurodevelopmental outcomes. Similar results (no association with worse outcomes after multivariable analysis) were found by Maiocco et al. [36] after an evaluation of preterm infants with a gestational age of less than 30 weeks using Griffiths Mental Developmental Scores (GMSD) at 24 months corrected gestational age. EUGR was defined in this study by a weight less than the 10th percentile using INTERGROWTH-21st. Studying the same population and this definition of EUGR with the same growth chart, contrary results were found by De Rose et al. [38], who observed a significant association with worse GSMD and Gross Motor Functional Classification System (GMFCS).

For the definition of EUGR as the weight at discharge using less than −2 or −1.5 z-scores, most of the studies did not find a consistent capability of EUGR to predict worse neurodevelopmental outcomes [20,27,29,32]. Zozaya et al. [20] evaluated VLBW infants of less than 34 weeks of gestational age with EUGR defined as a z-score less than −1.5 at 36 weeks of postmenstrual age and found no association with worse BSID-II at 24 months of corrected gestational age. Hack et al. [27] found no significant association with worse BSID-I and EUGR at 40 weeks of postmenstrual age in VLBW infants if catch-up had occurred by 8 months of gestational age. Tudehope et al. [29] did not find worse GMSD results at 3 years of life of VLBW infants if catch-up occurred. However, it is important to note that a study from 2021 found a significant association of a higher risk of cognitive delay and EUGR defined as a weight z-score at discharge below −1 [37]. Moreover, a study published in 2018 that classified preterm infants by the severity of EUGR considering a weight z-score at discharge <−2, <−2.5 y < −3 found a significant relation between RCEU and the risk of a mental developmental index (MDI) less than 85 at 24 months of corrected age, with an increasing risk with the increasing severity of EUGR (z < −2.5, OR: 1.92; z < −3.0, OR: 2.83) [34]. De Rose et al. [38] compared 12 cross-sectional EUGR definitions by weight using two growth charts (INeS and INTERGROWTH-21st), two weight cut-offs (10th centile and 2 standard deviations) and six different time-points. This article found a significant association with worse neurodevelopmental outcomes with all the cross-sectional criteria studied except a weight less than the 10th centile and less than 2 SDs at discharge using INeS.

Disparities in the predictive capacity of neurodevelopmental outcomes of cross-sectional EUGR persist later in childhood. While a study assessing a small cohort of infants with postnatal growth failure (defined by a weight at discharge less than the 3rd centile) reported worse scores of the Full-Scale Intelligence Quotient and some domains of the Wechsler Intelligence Scale for Children, Fourth Edition [40]; some studies have not found a significant association with adverse outcomes [32,39]. Kan et al. [32] evaluated the Wechsler Intelligence Scale for Children, Third Edition (WISC-III), Wide Range Achievement Test, Third edition (WRAT3) and Movement Assessment Battery for Children (Movement ABC) of infants with a gestational age of less than 28 weeks. They found no relation between weight and neurodevelopmental outcomes at 8 years of life. Of consideration, Alcantara et al. [39] did not find a significant association between EUGR and clinical neurological development disorder. The authors described that the Reynolds Intellectual Screening Test (RIST) index at 5–7 years correlated with the z-score weight at discharge with no correlation with the Developmental Neuropsychological Assessment, Second Edition (NEPSY-II) assessment.

### 3.2. Longitudinal EUGR and Neurodevelopmental Outcomes

Controversy persists with studies assessing neurodevelopmental outcomes in relation to longitudinal EUGR (weight loss more than a variable z-score cut-off at a specific point in time compared to birth), as outlined in Table 2.

As discussed earlier, variability is present in the longitudinal definition itself, with differences in z-score cut-offs and the timing of assessment used for the definition of EUGR among the reviewed articles. Moreover, substantial variability is observed in the methods performed to assess neurodevelopmental outcomes in the literature.

A relation of longitudinal EUGR and poorer neurodevelopmental outcomes early in infancy, at 18 to 24 months of corrected age, has been described in the literature [20,21,38,41,44,45,46]. Shah et al. [21] described a significant association of Z-score difference from birth more than 2 with the psychomotor developmental index (PDI) but not with the mental developmental index (MDI) in preterm infants of less than 28 weeks of gestational age. This association was not significant when the EUGR cut-off was set as a Z-score difference from birth more than one. Zozaya et al. [20] described that every 1-point fall in the weight z-score using Fenton was associated with a 5.6-point decrease in the MDI at 24 months of corrected gestational age in preterm infants of less than 34 weeks of gestational age. Frondas-Chauty et al. [41] studied infants of less than 33 weeks of gestational age using the weight z-score difference from birth to discharge, establishing different subcategories, and found that inefficient growth during hospitalization is associated with a non-optimal neurological outcome at 2 years of age. El Rafei et al. [46] evaluated infants of less than 32 weeks of gestational age using Fenton and described the increased risk of neurodevelopmental impairment with severe EUGR (unadjusted) and the increased risk in boys with severe EUGR (adjusted). Yitayew et al. [45] observed a significant association between growth failure (weight z-score decrease more than 1 SD from birth to discharge using Fenton and INTERGROWTH-21st) and poor neurodevelopmental outcomes using BSID-III at 12 and 24 months of corrected age.

Nevertheless, other studies have found no association between longitudinal EUGR and poor neurodevelopmental outcomes at 24 weeks of corrected gestational age after multivariate analysis [36,38,48]. Maiocco et al. [36] described no association with a fall in weight of more than 1 Z-score from birth to discharge using INTERGROWTH-21st and worse outcomes after multivariable analysis. Strobel et al. [48] studied preterm infants with gestational age from 24 to 28 weeks and found no significant association after adjustments for comorbidities with a weight z-score decrease more than 0.8 z-score from birth to discharge and worse BSID-III at 20–33 months.

De Rose et al. [38] compared 12 longitudinal EUGR definitions by weight using two growth charts (INeS and INTERGROWTH-21st), two weight z-score decrease cut-offs (loss of 1 and 2 standard deviations) and six different time-points. A significant association with EUGR and worse neurodevelopmental outcomes was found with a decrease in the weight z-score more than 1 SD from 2 weeks after birth or at 27 weeks cGA to discharge or 36 weeks of cGA using INeS. No significant association was found with any of the criteria using INTERGROWTH-21st or any of the definitions using different time-points from birth with INeS or INTERGROWTH-21st. This study suggests a better prediction of neurodevelopmental outcomes using the criteria of a loss of more than 1 SD in weight, calculated after physiological weight loss and identified as soon as possible rather than at discharge.

It is also important to acknowledge the conflicting results reported when assessing neurodevelopment with the mental developmental index (MDI) and psychomotor developmental index (PDI) as previously described. While Shah et al. reported a significant association of the Z-score difference from birth >2 with the PDI but not with the MDI [21], Zozaya et al. described that every 1-point fall in the weight z-score was associated with a 5.6-point decrease in the MDI [20], and Cordova et al. described the association of EUGR with an MDI < 85 [44].

Increasing divergence in the assessed studies has been described when evaluating the ability of longitudinal EUGR to predict neurodevelopmental outcomes later in childhood. Cordova et al. described the association from EUGR with Fenton and Olsen charts with low neurodevelopmental scores but not with INTERGROWTH-21 at 7 years of corrected gestational age [44]. El Rafei et al. [35] described lower IQ in children at 5 years of age who had EUGR without cerebral palsy (−3.9 points, 95% Confidence Interval (CI) = −7.2 to −0.6 for Fenton) [47]. However, these authors found no association between longitudinal EUGR and motor function and cerebral palsy [47]. Contrarily, Leppänen et al. [42], Kan et al. [32] and Alcántara et al. [39] did not find an association with adverse neurodevelopmental outcomes at 5, 5–7 and 8 years, respectively.

### 3.3. “True” EUGR and Neurodevelopmental Outcomes

Compared to cross-sectional and longitudinal definitions, fewer studies have investigated the relation between “true” EUGR and neurodevelopmental outcomes, as described in Table 3. Consistent with the previously described articles, heterogeneity is observed in the methods performed to assess neurodevelopment.

Among the reviewed studies, two evaluated “true” cross-sectional EUGR at discharge. Ramel et al. found no association between neurodevelopmental outcomes at 24 months of corrected age and weight z-scores at discharge in a cohort of appropriate for gestational age (AGA) infants described as having a birth weight between the 3rd and 97th percentile using Fenton charts [49]. Alcántara et al. did not identify a significant relation between clinical neurodevelopmental disorders at 5–7 years and EUGR for both Fenton and INTERGROWTH-21 [40]. We have also included a study from Guellec et al. [50] that evaluated extrauterine growth defined by weight gain or loss between birth and 6 months by the z-score change. This study described catch-down growth as AGA at birth with a z-score difference ≥−1 SD from birth to 6 months and an observed greater risk of cerebral palsy being for AGA children who experienced catch-down (stratified OR 2.26, 95% CI [1.37–3.72]). It also described higher rates of inattention–hyperactivity symptoms, moderate-to-severe cognitive deficiency and difficulties in school, but those did not persist when adjusted in a multivariate analysis.

### 3.4. EUGR by Length and Head Circumference and Neurodevelopmental Outcomes

A group of experts published in 2020 an article highlighting that EUGR is not usually defined by length or head circumference [25]. Similar to as previously described with weight assessment, significant variability is noted in studies evaluating EUGR by head circumference or length with different criteria used and different neurodevelopmental assessments. Moreover, conflicting results have also been reported.

Several studies have described a significant association between head growth impairment and worse neurodevelopmental outcomes [38,39,42]. De Rose et al. [38] compared 24 EUGR definitions by head circumference using two growth charts (INeS and INTERGROWTH-21st), two head circumference z-score decrease cut-offs (loss of 1 and 2 standard deviations) and six different time-points. They described higher cognitive scores and subscale evaluations in infants whose head circumference Z-scores did not decrease by one or more SDs from 2 weeks of age (or from 27 weeks postmenstrual age) to age 36 weeks PMA (or discharge, if earlier). This study suggests a better prediction of neurodevelopmental outcomes using the criteria of a loss of more than 1 SD in head circumference, calculated after physiological weight loss and identified as soon as possible rather than at discharge. Maiocco et al. [36] evaluated EUGR by head circumference in preterm infants with a gestational age of less than 30 weeks. The authors defined EUGR as a measurement less than the 10th percentile at discharge (cross-sectional), a decrease in the z-score of more than 1 from birth to discharge (longitudinal) and a decrease in the head circumference z-score between 14–21 days of life and discharge (new longitudinal “post-loss”). A significant association between the head circumference z-score at birth and minor impairment was also described in this study. After an adjustment for the confounding variables, only the longitudinal post-loss definition maintained a statistically significant predictive value. Alcántara et al. [39] observed the correlation of the Reynolds Intellectual Screening Test (RIST) score and the head circumference z-score at birth with Fenton. Nevertheless, it is important to note that this study found no significant correlation with the head circumference z-score at birth using INTERGROWTH-21st or the z-score at discharge using Fenton or INTERGROWTH-21st or with z-score differences between birth and neonatal discharge. Leppänen et al. [42] evaluated infants with a gestational age of less than 32 weeks or a birth weight less than 1501 grams. This study describes nonsignificant correlations between head circumference z-score changes from birth to 36 weeks and 40 weeks of corrected gestational age and full-scale IQ (FSIQ) results at 5 years old. However, the authors found a statistically significant correlation between the previously presented parameters when analyzing the subgroup of infants who were not small for their gestational age. Moreover, a systematic review has also reported positive associations between postnatal head growth and neurocognitive outcomes [51]. This review described generally consistent associations of postnatal head growth and neurocognitive outcomes measured at ages ranging from 12 months old to adulthood.

Nevertheless, other studies, as previously described with Alcantara et al. [39] or Strobel et al. [48], have described no association of EUGR with head circumference and worse neurodevelopmental outcomes. The latest defined head circumference as a z-score decrease from birth to discharge of more than 0.8 SDs, with no association with BSID-III cognitive, motor or language scores [48].

Conflicting results have also been described regarding growth assessment by length and neurodevelopmental impairment. Some studies have found no significant association [42] while others have found a significant association between poor linear growth and neurodevelopmental outcomes [39,44] as well as higher cognitive scores with accelerated linear growth from birth to discharge [48]. Leppänen et al. [42] described nonsignificant correlations between length z-score changes from birth to 36 weeks and 40 weeks of corrected gestational age and full-scale IQ (FSIQ) results at 5 years old. Regarding the studies with positive associations, Alcántara et al. [39] observed statistically significant correlations between the RIST test and the length z-score at birth and discharge using Fenton and INTERGROWTH-21st. No significant correlation was found between the RIST index and the z-score difference between birth and neonatal discharge nor with the Developmental Neuropsychological Assessment, Second Edition, (NEPSY-II). Cordova et al. [44] observed that poor linear growth, described as a decline of more than 2 SDs in length from birth to term equivalent age, was associated with a worse verbal intelligence quotient at 7 years of age. Nevertheless, it is important to recognize that this association was found using the Olsen reference but not with INTERGROWTH-21st. Strobel et al. [48] studied preterm infants with a gestational age from 24 to 27 weeks and 6 days using Fenton. This study described the association of accelerated linear growth, defined as a z-score increase in length from birth to discharge of more than 0.8 SDs, with increased BSID-III cognitive scores after adjustment. No association was found with BSID-III motor or language scores.

Given the potential utility of length and head circumference, the study by the group of experts presented earlier [25] suggests consideration should be given to the three parameters (weight, length and head circumference) for the anthropometric evaluation of the preterm infant. They suggested that these measurements should be taken regularly (at least weekly) throughout the admission.

## 4. Conclusions

In this paper, we have reviewed EUGR by weight, including cross-sectional, longitudinal and “true” EUGR definitions, and the potential relation between these concepts and adverse neurodevelopmental outcomes in preterm infants.

We have highlighted the challenges to compare different published studies on the subject, even when subclassifying by the definition of EUGR, due to the significant variability in the criteria used for each definition and in the tests used for the evaluation of neurodevelopmental outcomes in the different papers reviewed. This heterogeneity compromises the obtention of a single firm conclusion on the relation between EUGR by weight and adverse neurodevelopmental outcomes of the review due to the impossibility of directly comparing most of the reviewed articles with each other.

The further refinement and clarification of these concepts would be essential to gain deeper insights into EUGR implications in neurodevelopment in preterm infants.

## Figures and Tables

**Table 1:** Summary of studies assessing the cross-sectional definition *.

Study	Year	EUGR Definition	Growth Chart	Population	Neurodevelopmental Assessment	Outcomes
Hack et al. [27]	1982	Weight z-score < −2 at 40 weeks	Babson and Benda [28]	192 VLBW infants.	BSID-I of neurosensory impairment at 8 months.	No significant association if catch-up occurred by 8 months of corrected age.
Tudehope et al. [29]	1983	Weight z-score < −2 at discharge	Usher–McLean Intrauterine Growth Chart [30]	164 VLBW infants.	GMSD at 3 years.	EUGR not predictive of neurodevelopmental outcome if catch-up occurred.
Shah et al. [21]	2006	Weight < 10th centile and <3rd centile at 36 weeks	Kramer [31]	221 infants, ≤28 weeks gestational age (GA).	BSID-II at 18–24 months or best clinical estimate of performance.	No significant association with neurodevelopmental outcomes.
Kan et al. [32]	2008	Weight z-score at discharge	Cole [33]	401 infants, <28 weeks of GA.	WISC-III, WRAT3 and movement ABC at 8 years.	Weight not related to outcomes.
Chien et al. [34]	2018	Weight z-score < −2, <−2.5 and <−3 at discharge	Hsieh [35]	224 VLBW infants.	BSID-II at 24 months cGA.	EUGR associated with MDI < 85, and this association was related to the severity of EUGR.
Zozaya et al. [20]	2018	Weight z-score < −1.5 at 36 weeks	Fenton	168 VLBW infants, <34 weeks of GA.	BSID-II at 24 months cGA.	No association with worse BSID-II.
Maiocco et al. [36]	2020	Weight < 10th percentile at discharge	INTERGROWTH-21st	195 infants <30 weeks of GA.	GMSD at 24 (+−6) months cGA.	No association with worse outcomes after multivariable analysis.
Salas et al. [37]	2021	Weight Z-score < −1 at 36 weeks cGA	INTERGROWTH-21st	359 infants 24–26 weeks of GA.	BSID-III at 24 months cGA.	Significant association with higher risk of cognitive delay.
De Rose et al. ** [38]	2021	Weight < 10th percentile at discharge or 36 weeks cGA. Weight Z-score <−2 SDS at discharge or 36 weeks	Italian neonatal study charts (INeS) and INTERGROWTH-21st	254 infants ≤30 weeks.	GMSD at 24 (+−4) months cGA and GMFCS.	Significant association with EUGR definitions by <10th percentile and <−2 z-score and worse GSMD and GMFCS.
Alcántara et al. [39]	2021	Weight < 10th percentile at discharge.	Fenton and INTERGROWTH-21st	87 VLBW infants.	RIST and NEPSY-II at 5–7 years	No significant association between EUGR and clinical neurological development disorder.
Kim et al. [40]	2023	Weight < 3rd percentile at discharge.	Fenton	82 infants, 21 VLBW with EUGR.	MRI, K-WISC-IV, KEDI-WISC, ATA and executive function at 6–8 years.	Infants with EUGR had significantly lower FSIQ scores and 3 index scores in K-WISC-IV. Higher ATA score (worse function) with EUGR.

* Abbreviations used in Table 1: Very-low birth weight (VLBW), Bayley Scales of Infant Development, First, Second and Third Edition (BSID-I, BSID-II and BSID-III, respectively), Griffiths Mental Developmental Scores (GMSD), Gross Motor Functional Classification System (GMFCS), Wechsler Intelligence Scale for Children, Third Edition (WISC-III), Wide Range Achievement Test, 3rd edition (WRAT3), Movement Assessment Battery for Children (Movement ABC), mental developmental index (MDI), RIST test (Reynolds Intellectual Screening Test), NEPSY-II (Developmental Neuropsychological Assessment, Second Edition), Magnetic resonance imaging (MRI), Korean version of the Wechsler Intelligence Scale for Children, Fourth Edition (K-WISC-IV) and the Korean Educational Development Institute–Wechsler Intelligence Scale for Children (KEDI-WISC), Advanced Test of Attention (ATA), and Full-Scale Intelligence Quotient (FSIQ); ** De Rose et al. [38] compared 12 cross-sectional definitions of EUGR by weight using different time-points. In the table, only definitions using the most common criteria have been summarized.

**Table 2:** Summary of studies assessing the longitudinal definition *.

Study	Year	EUGR Definition	Growth Chart	Population	Neurodevelopmental Assessment	Outcomes
Shah et al. [21]	2006	Weight z score difference > 1 and >2 from birth to 36 weeks.	Kramer	221 infants, ≤28 weeks gestational age (GA).	BSID-II at 18–24 months or best clinical estimate of performance.	Significant association of Z-score difference from birth >2 with PDI but not with MDI. Not significant for Z-score >1.
Kan et al. [32]	2008	Weight z-score change from birth to discharge	Cole	401 infants, <28 weeks of GA.	WISC-III, WRAT3 and movement ABC at 8 years.	Weight not related to outcomes.
Frondas-Chauty et al. [41]	2014	Weight z-score difference from birth to discharge (<−2, −2 to −1.01, −1 to −0.51, −0.50 to 0.01 and ≥0, the reference).	Olsen for infants discharged <41 weeks, WHO for infants discharged >41 weeks.	2047 infants, <33 weeks of GA.	Physical exam, PY-BL-R and ASQ at 24 months.	Inefficient growth during hospitalization is associated with a non-optimal neurological outcome at 2 years of age.
Leppänen et al. [42]	2014	Weight z-score change from birth to 36 and 40 weeks.	Sorva [43]	274 infants, <1501 g or less than 32 weeks of GA.	WPPSI-R at 5 years.	No association with 5-year cognitive outcome.
Zozaya et al. [20]	2018	Fall in weight z-scores from birth to 36 weeks.	Fenton	168 VLBW infants, born <34 weeks of GA.	BSID-II at 24 months cGA.	Every 1-point fall in weight z-score was associated with a 5.6-point decrease in the MDI.
Cordova et al. [44]	2020	Weight z-score decline > 0.8 SD from birth to term-equivalent.	Fenton, Olsen and INTERGROWTH-21st	613 infants, <33 weeks of GA.	BSID-II at 18 months corrected age. WASI test and WRAT4 at 7 years of corrected age.	EUGR with Fenton and Olsen was associated with low neurodevelopmental scores. EUGR with Fenton was associated with MDI < 85.
Maiocco et al. [36]	2020	Fall in weight z-score > 1 from birth to discharge	INTERGROWTH-21st	195 infants < 30 weeks of GA.	GMSD at 24 (+−6) months cGA	No association with worse outcomes after multivariable analysis
Yitayew et al. [45]	2021	Weight z-score decrease > 1 from birth to discharge.	Fenton and INTERGROWTH-21st	340 preterms, <33 weeks of GA.	BSID-III at 12 and 24 months of corrected age.	Significant association between growth failure and poor neurodevelopmental outcomes.
De Rose et al. ** [38]	2021	Weight z-score decrease > 1 from 2 weeks after birth or at 27 weeks cGA to discharge or 36 weeks of cGA	Italian neonatal study charts (INeS) and INTERGROWTH-21st	254 infants ≤ 30 weeks.	GMSD at 24 (+−4) months cGA and GMFCS.	Association with worse GSMD and GMFCS using INeS but not significant with INTERGROWTH-21st
Alcántara et al. [39]	2021	Weight z-score difference from birth >1 or >2 from birth to discharge.	Fenton and INTERGROWTH-21st	87 VLBW infants.	RIST and NEPSY-II at 5–7 years.	No significant association between EUGR and clinical neurological development disorder.
El Rafei et al. [46]	2021	Weight z-scores difference < −2 (severe) and −2 to −1 (moderate) from birth to discharge.	Fenton	4197 infants, <32 weeks of GA.	Standardized parental questionnaire at 24 months.	Increased risk of neurodevelopmental impairment with severe EUGR (unadjusted). Increased risk with boys with severe EUGR (adjusted).
El Rafei et al. [47]	2023	Weight z-scores difference < −2 (severe) and −2 to −1 (moderate) from birth to discharge.	Fenton	957 infants, <28 weeks of GA.	CP diagnosis, WPPSI-R and Movement ABC-2 at 5 years.	Severe EUGR related to lower IQ. No significant associations were observed between motor function and CP.
Strobel et al. [48]	2024	Weight z-score decrease ≥ 0.8 from birth to discharge.	Fenton	590 infants in preterms 24 to 27 + 6 weeks GA.	BSID-III at 20–33 months. CBCL at 1–5 years.	No significant association after adjustments for comorbidities.

* Abbreviations used in Table 2: Bayley Scales of Infant Development, Second Edition (BSID-II), mental developmental index (MDI), psychomotor developmental index (PDI), Wechsler Intelligence Scale for Children-Third Edition (WISC-III), Wide Range Achievement Test, 3rd edition (WRAT3), Movement Assessment Battery for Children (Movement ABC), Wechsler Preschool and Primary Scales of Intelligence–Revised (WPPSI-R), Wechsler Abbreviated Scale of Intelligence (WASI), Wide Range Achievement Test, fourth edition (WRAT4), Griffith’s Mental Developmental Scores (GMSD), Gross Motor Functional Classification System (GMFCS), Bayley Scales of Infant Development, Third Edition (BSID-III), Reynolds Intellectual Screening Test (RIST test), Developmental Neuropsychological Assessment, Second Edition (NEPSY-II), cerebral palsy (CP), intelligence quotient (IQ), Child Behavior Checklist (CBCL); ** De Rose et al. [38] compared 12 longitudinal definitions of EUGR by weight with different time-points. In the table, only definitions using the most common criteria have been summarized.

**Table 3:** Summary of studies assessing “true” EUGR definition *.

Study	Year	EUGR Definition	Growth Chart	Population	Neurodevelopmental Assessment	Outcomes
Ramel et al. [49]	2014	AGA at birth, weight z-score at discharge.	Fenton	62 AGA, ≤30 weeks GA.	BSID-III at 24 months of corrected age.	Weight z-score at discharge (when length and head circumference z-score were controlled for) was not associated with 24-month cognitive scores.
Guellec et al. [50]	2016	AGA at birth with weight z-score difference ≥−1 from birth to 6 months.	WHO	1493 infants, <32 weeks of GA.	Medical examination, K-ABC and behavioral difficulties at 5 years. School performance at 8 years.	Higher risk of cerebral palsy. No other significant differences in outcomes after adjustment on multivariate analysis.
Alcántara et al. [39]	2021	Not IUGR infants with weight at discharge < 10th percentile.	Fenton and INTERGROWTH-21st	87 VLBW infants.	RIST and NEPSY-II at 5–7 years.	No significant association between EUGR and clinical neurological development disorder.

* Abbreviations used in Table 3: appropriate for gestational age (AGA), Very-low birth weight (VLBW), Bayley Scales of Infant Development III (BSID-III), Kauffman Assessment Battery for Children (K-ABC), RIST test (Reynolds Intellectual Screening Test), and NEPSY-II (Developmental Neuropsychological Assessment, Second Edition).
